# Journal Club in Residency Education: An Evidence-based Guide to Best Practices from the Council of Emergency Medicine Residency Directors

**DOI:** 10.5811/westjem.2018.4.37507

**Published:** 2018-05-15

**Authors:** Michael Gottlieb, Andrew King, Richard Byyny, Melissa Parsons, John Bailitz

**Affiliations:** * Rush University Medical Center, Department of Emergency Medicine, Chicago, Illinois; † The Ohio State University Wexner Medical Center, Department of Emergency Medicine, Columbus, Ohio; ‡ Denver Health Medical Center, Department of Emergency Medicine, Denver, Colorado; § University of Florida College of Medicine – Jacksonville, Department of Emergency Medicine, Jacksonville, Florida; ¶ Northwestern University Feinberg School of Medicine, Department of Emergency Medicine, Chicago, Illinois

## Abstract

Journal clubs are an important tool for critically appraising articles and keeping up-to-date with the current literature. This paper provides a critical review of the literature on the design and structure of journal clubs in residency education with a focus on preparation, topic selection, implementation, and integration of technology. Recommendations for preparation include developing clearly defined goals and objectives that are agreed upon by all journal club participants; mentorship from experienced faculty members to ensure appropriate article selection, maintenance of structure, and applicability to objectives; distribution of articles to participants 1–2 weeks prior to the scheduled session with reminders to read the articles at predetermined intervals; and the use of a structured critical appraisal tool for evaluating the articles. Recommendations for topic selection include selecting a primary objective of either critical appraisal or informing clinical practice and ensuring that the articles align with the objective; involving learners in the topic- and article-selection process; and having the article selection driven by a specific clinical question. Recommendations for implementation include hosting sessions in the evening and away from the hospital environment; providing food to participants; hosting meetings on a monthly basis at regularly scheduled intervals; mandating journal club attendance; and using theories of adult learning. Recommendations for integration of technology include using previously established, effective strategies and determining the feasibility of creating an online journal club versus joining an established journal club. It is the authors’ intention that after reading this paper readers will have new strategies and techniques for implementing and running a journal club at their home institutions.

## BACKGROUND

While the concept of the journal club is most commonly attributed to Cushing’s description of Sir William Osler’s meetings in 1875, the first reference dates back to 1835 when Sir James Paget would meet with a group of students near St. Bartholomew’s Hospital to review articles.^1^–^3^ Initially, the journal clubs served as a method “for the purchase and distribution of periodicals to which [members] could ill afford to subscribe as an individual.”^1^ As time progressed, the focus expanded to serve as a medium to teach critical appraisal skills.^4^,^5^

While many programs use journal clubs in their graduate medical education training, there is significant variation in the structure and goals.^6^,^7^ Additionally, success can vary with wide ranges in attendance, participation, and longevity.^4^ It is important to use effective strategies to increase the likelihood of creating and maintaining a successful journal club. This article provides a narrative summary of the literature and recommendations for best practices for journal clubs in graduate medical education with a focus on the application to emergency medicine (EM) residency programs.

### Critical Appraisal of the Literature

This article is the first in a series of evidence-based best practice reviews from the Council of Emergency Medicine Residency Directors (CORD) Best Practices Subcommittee. The first two authors independently performed a search of PubMed for articles published from inception to August 20, 2017, using the keywords “journal club.” Bibliographies of all relevant articles were reviewed for additional studies. The search was further augmented by several calls via social media to the #FOAMed and #MedEd communities requesting additional article recommendations. Articles were screened independently by two authors to evaluate for any papers addressing the following four themes, which were determined a priori: preparation for journal club; topic selection; strategies for successful implementation; and incorporation of technology. Articles were included if either author recommended inclusion.

The search yielded a total of 2,102 articles, of which 67 were deemed to be directly relevant for inclusion in this review. When supporting data was not available, recommendations were made based upon the authors’ combined experience and consensus opinion. Level of evidence was provided for each statement according to the Oxford Centre for Evidence-Based Medicine criteria (Table 1).^8^ Prior to submission, the manuscript was reviewed by the entire CORD Best Practices Subcommittee.

### Preparation for Journal Club

Successful journal clubs are predicated upon thorough preparation and the development of clear, specific goals and objectives. Defining and articulating the goals and objectives of any educational experience is an important pedagogical step;^4^ in fact, developing clear goals and objectives has been suggested to be the first and most important step in the creation of a successful journal club.^9^–^11^ Reflection on the defined goals will guide further decisions regarding journal club format, the selection of facilitators, and the types of articles to review. Goals should be reviewed regularly and approved by journal club participants.^10^ Explicit statement of the goals, creation of learning objectives, and selection of the most appropriate session format were all found to be factors that increase the educational benefit among journal club participants.^4^,^11^

Several surveys across multiple medical specialties have assessed the most common goals for a journal club. These include teaching critical appraisal skills, providing an impact on clinical practice, remaining current on medical literature, allowing residents and faculty to work together on a common project, and learning research methodology.^4^,^6^,^12^–^14^ Among these, teaching critical appraisal skills was considered the most important goal for journal clubs. In their recommendations for journal club implementation, Lee and colleagues have defined a set of objectives for teaching and assessing practice-based learning, which is illustrated in Table 2.^15^ In his paper describing the role of journal clubs in orthopedic residencies, Greene identified similar goals to those defined by Lee; however, he added the benefits of residents to learn a specialty and the development of camaraderie between residents and faculty.^16^

Another important aspect is support and mentorship from more experienced faculty members in the form of advice and technical support to resident physicians. Mentorship includes assistance in the selection of an article for critical appraisal and preparing the associated presentation.^16^ Similarly, faculty mentors should prepare the residents to lead the discussion by both ensuring the mastery of the chosen article’s critical appraisal and planning the interactive discussion. This supportive approach ensures maintenance of a consistent format and systematic presentation without restricting resident creativity.^18^ When in-person mentorship is not feasible, Kattan and colleagues have advocated for journal club leaders to receive telephone- or email-based coaching instead.^19^ Learners were coached on enhancing their discussion leadership skills, while focusing on the standard journal club aims of critical reading, interpretation, and developing content knowledge. Additionally, coaches assisted the learners with the development of structured outlines, effective teaching strategies, and visual aids for the journal club session.^19^

Studies unanimously agree that selected articles should be distributed to all members of the journal club prior to the session. A systematic review by Deenadayalan and colleagues found that the preparation time for those who attended journal clubs varied widely and recommended a minimum of one week.^20^ Subsequent studies similarly recommend at least a one-week preparatory period, while the maximum recommended time period was two weeks.^10^,^11^,^18^,^21^–^23^ It has been suggested that using a time period longer than two weeks can lead to the participant forgetting what they read before the journal club session.^10^

When participants review articles prior to the journal club, article evaluation guidelines or a critical appraisal instrument should be used. This practice has been consistently reported in the literature as a feature of successful journal clubs.^4^,^9^–^11^,^15^–^18^,^20^–^25^ However, Carpenter and colleagues found that most EM programs (71%) do not use structured critical appraisal instruments.^7^ The use of critical appraisal checklists have been shown to increase learner satisfaction, improve the educational value of the journal club, and promote productive discussion, without increasing the overall workload.^20^,^21^,^26^ These instruments can have many formulations, ranging from a list of questions to a structured worksheet^10^,^27^ (Figure).

**Best Practice Recommendations:**

1. Develop clearly defined goals and objectives that are agreed upon by all journal club participants (Level 2a).
2. Faculty members or more experienced clinicians should provide mentorship to ensure appropriate article selection, maintenance of structure, and applicability to objectives (Level 2a).
3. Distribute articles to participants one to two weeks prior to the scheduled session and consider reminders to read the articles at predetermined intervals (Level 4).
4. Use a structured critical appraisal tool when evaluating articles for journal club (Level 2a).

### Topic Selection

The first consideration that should be made when selecting journal club topics are the overall objectives.^4^ In fact, many authors make a distinction between a journal club and an evidence-based medicine session. Journal clubs are typically described as sessions focused on reviewing articles to inform clinical practice, while evidence-based medicine sessions focus on learning the skills to critically appraise the articles.^17^

This distinction in goals may influence the topic selection methods used for the session. For example, a journal club session with a primary objective of teaching principles of evidence-based medicine might select very different articles to review than one with a primary goal of keeping participants informed on the most current literature. Alternatively, topic selection techniques focused on enhancing resident participation (e.g., having learners select the article to discuss) may result in different learner goals, with variations in the applicability to practice or the ability to teach critical appraisal skills. Moreover, the curriculum and the impact of the selected topic may be influenced by the experience of the learner. Harris and colleagues highlighted that “a [journal club] for students or interns may include the same ingredients, but in different proportions, with more emphasis on learning the ‘rules’ of critical appraisal and the topics of clinical epidemiology and biostatistics.”^17^ Additional considerations are included in Table 3.

As a result, some experts have suggested separating the curriculum that teaches skills in epidemiology from the curriculum that reviews the newest literature. One study found that this separation improved the ability of participants to acquire the skills and knowledge in each area.^17^ The authors from this study further suggested that having a baseline clinical knowledge on a topic can be helpful as the learners will not need to familiarize themselves with the content, allowing them to focus predominantly on skill development.^17^ Another study found that less-experienced learners had more difficulty with critical appraisal because they were focused on mastering the content rather than critical analysis.^28^ To balance this, one study divided up the journal club sessions, so that some were focused on specific topics, while others were focused on critical appraisal and methodology.^29^

When selecting article topics, it can be valuable to begin with a specific clinical question. These may be based upon actual cases encountered by the learner, hypothetical cases, or new literature. The use of a particular case or challenge may increase learner interest and engagement. Several authors have described the importance of a clinical problem-based article selection, though formal comparative studies are limited.^4^,^20^,^30^–^33^

In a systematic review on journal clubs published by Honey and colleagues, nine of the 14 included articles had the participants select the topic.^21^ Adult learning theories suggest that by having the learner drive the selection of the material there may be better engagement in the discussion process.^33^–^35^ In fact, one study found that the active participation of the learners in the planning, preparation, and facilitation of the session was associated with higher attendance and better overall success.^9^ Learners also benefit by learning the critical steps in translating a clinical question into a query that can be used to search evidence databases.^36^,^37^ They can learn how to use formal search databases to access information and select the articles best suited to answer their question.^36^,^37^

It is important that the article search be performed in a structured manner to be effective. When performing a search for potential articles, learners first need to understand how to convert a clinical question into a query. They should receive structured training to be familiar with the variety of literature repositories (e.g., PubMed, Scopus, CINAHL). They should learn search strategies, such as using Boolean language and how to use MeSH terms. Training should include practice in searching article databases and feedback on their search strategy. Additionally, learners should receive instruction on which journals publish the highest quality literature in EM. Therefore, they often need to have direct mentorship in article selection techniques.^18^,^35^ One study found that faculty mentorship for article selection significantly improved participant satisfaction.^38^ Alternatively, faculty may select the articles for inclusion. This technique offers the advantage of ensuring high-quality article selection, while allowing the learners to focus on analyzing and applying the selected articles. However, this reduces the learners’ opportunity to develop their skills in query generation, database search strategies, or article selection. Interestingly, one study used a committee of both residents and attending physicians to assist with topic selection for their journal clubs with good learner satisfaction.^29^

It is important to vary the types of studies included to ensure that the learner develops the skills to analyze multiple different types of articles (e.g., retrospective, prospective observational, randomized controlled trial, systematic review and meta-analysis);^35^ however, the structure of this may vary depending upon the session goals. For example, if the session goal is to critically analyze the performance of chart review studies, then only retrospective chart reviews will be included, while if the focus is on a particular topic, then multiple article types may be valuable. This can allow the learners to compare and contrast the different article formats.^35^ While the ideal number of papers to review has not been formally studied, it is important to ensure a balance of topic breadth with depth. Selecting more articles may increase the potential yield in a broader sense, by covering more material, at the expense of reducing the ability to perform in-depth analyses.^35^ Two large surveys both found that the majority of programs assessed between one and four articles at each session.^39^,^4^

**Best Practice Recommendations:**

1. Determine whether the primary objective is critical appraisal or informing clinical practice and ensure that the articles selected align with this objective. While these are not mutually exclusive, one often predominates (Level 4).
2. Involve learners in the topic and article selection process to increase learner engagement and maximize the learner benefit (Level 3b).
3. When possible, have the article selection driven by a specific clinical question (Level 3a).
4. Article selection and topic selection should have active mentorship from a faculty member (Level 4).

### Implementation Strategies

While there are numerous studies assessing different aspects of journal clubs, there is no standard process for how to implement a journal club. Review of the existing literature demonstrated that the setting of a journal club varied significantly between studies, including conference rooms, faculty members’ houses, restaurants, and the hospital. The timing also varied in the studies, with some meeting in the evening, while others met during work hours. This variation is likely dependent upon the physician type, due to the variations in schedules and work hours. For example, a study conducted in an internal medicine residency program, found meeting during the lunch hour to be the most common.^39^ Similarly, a survey of surgical residency programs indicated they were split between morning (29%), midday (29%) and evening (42%),^40^ while a study of anesthesiology resident physicians found that 53% preferred to meet before work and 40% preferred to meet after work, with 57% preferring the workplace.^41^ Unfortunately, this is often not feasible with EM resident physician schedules. When studied within EM, successful journal clubs were most commonly held in the evening in a faculty member’s home.^4^,^42^ Jouriles performed a large survey of EM residency programs and discovered that 32% of programs scheduled journal club during didactic sessions, while the majority occurred outside of conference time.^6^ In that study, journal club was found to be most successful when it was held in the evening, outside of conference, and at the home of a faculty member.^6^

Although there are variations in the time and place of hosting a journal club, one of the most significant factors associated with successful journal clubs was the availability of food as an incentive.^10^,^16^,^20^,^22^,^39^,^41^ Regardless of the location or timing, the availability of food has been associated with increased longevity of the journal club (>2 years) and higher attendance.^4^,^38^ Studies have found benefit regardless of the quantity of food, with some providing light refreshments while others provide full dinners.^10^,^16^,^20^,^22^,^39^,^41^

The optimal frequency of journal club meetings has not yet been established. Meeting too often may result in lower attendance, while meeting infrequently may decrease the retention of evidence-based medicine concepts. Most programs meet every two to four weeks for journal club sessions.^16^,^19^–^21^,^39^–^43^ Survey data has found that monthly sessions are most common among surgery (64%),^40^ orthopedic surgery (78%),^16^ anesthesiology (70%),^41^ family practice (81%),^43^ and EM (86%) residency programs.^6^ Among internal medicine programs, there was more variation with 42.7% meeting monthly, 28.2% meeting bi-weekly, and 20.2% meeting weekly.^39^ Regardless of the specific frequency, it is important that the journal clubs occur in regular, predictable intervals and at the same time so that participants can anticipate and schedule accordingly. Several programs have demonstrated that set days and times promote attendance.^10^,^15^,^44^ Sadeghi found that redesigning journal club with an emphasis on regularity by predefining the entire schedule for the year was associated with increased resident satisfaction and improved self-assessments of evidence-based medicine knowledge.^29^

Most journal club sessions range from one-to-two hours in length.^16^,^19^,^21^,^39^,^40^ Among general surgical residency programs, 88% of journal clubs lasted one to two hours,^40^ while 83% of internal medicine programs lasted one hour and 95% lasted less than two hours.^39^ Among orthopedic surgery residency programs, 99% of journal clubs were between one-to-two hours in length.^16^ There is no data on session lengths specifically among EM residency programs, though it would be reasonable to extrapolate the above data to this field.

Another important consideration is whether the journal club sessions should be mandatory. In a survey of anesthesiology residents, 63% of residents preferred voluntary attendance.^41^ However, when evaluating successful journal clubs, mandatory attendance was one of the primary factors associated with success.^4^ Over half of all programs in multiple specialties have a mandatory attendance for journal club; however, the mean attendance is typically 60% for many programs.^39^,^40^,^43^ This may be due to a number of challenges, including clinical shifts, vacation, and external obligations. Deenadayalan proposed having regular journal club attendance be an expectation with the consideration of making it mandatory.^20^

While mandatory attendance will increase the number of learners, it is equally important to ensure that the sessions align with sound educational principles. First, the learners must feel that they are in a safe learning environment. The journal club must establish an environment that is conducive to learning, which includes learners being comfortable with their own limitations, as well as feeling comfortable discussing the specific limitations of a study.^21^ A safe learning environment will facilitate discussion and place learning in context.^23^,^45^ One study found that a less threatening, more egalitarian environment in journal club was of high value to learners to avoid the hierarchical nature inherent in residency and to facilitate the participation of all learners.^46^ Specifically, junior learners did not feel as comfortable discussing their opinions due to fewer clinical experiences.^46^ As a result, the authors suggested that there should be defined opportunities for more junior learners to contribute and efforts should be made to ensure all members feel included.^46^

Additionally, it is valuable to incorporate adult learning theories when designing journal club sessions (Table 4).^34^ As addressed previously, sessions should be focused on active involvement of participants from article selection to analysis, with an emphasis on incorporation of prior experiences and applicability to clinical cases.^4^,^7^,^9^,^17^,^30^ Studies have found that using multifaceted approaches to learning and integrating the education with clinical activities are associated with increased learner satisfaction and critical appraisal skills.^17^,^47^ The journal club format often includes components of one-on-one mentoring, formal presentation, and large-group discussion.^17^ Other approaches include a formal debate, written critique, discussion of research developments, small-group discussions, and competitions for best presentation.^4^,^6^,^22^,^48^,^49^ The use of gamification approaches, such as a debate or competition, may further increase user engagement, motivation, and participation in journal clubs.^49^–^51^

**Best Practice Recommendations:**

1. Sessions should be hosted in the evening and away from the hospital environment (Level 3b).
2. Food should be provided to participants (Level 3b).
3. Monthly meetings at regularly scheduled intervals are optimal for continued involvement of the residents and retention of evidence-based medicine concepts (Level 3a).
4. Journal club attendance should be mandated by program leadership (Level 3a).
5. Principles of adult learning theory should be upheld (Level 3b)

### Incorporation of Technology

Modern social media platforms (e.g., Twitter, Facebook, YouTube, blogs) provide the opportunity to readily create and participate in online journal clubs.^52^–^56^ Online journal clubs may help to improve journal club participation by removing barriers, such as time, location, and limited, local membership.^57^ Educators, content experts, investigators, and learners can all join in the discussion real-time (i.e., synchronous) or afterward (i.e., asynchronous) depending upon the platforms used.^56^ For educators interested in leveraging social media to accelerate the speed of knowledge translation at and beyond their institution, there are numerous papers across specialties that describe how to effectively create and conduct sustainable online journal clubs.^53^,^55^,^56^,^58^–^60^

As technology has evolved, so has the online journal club experience. Early journal clubs consisted of static webpages with experts providing summaries of articles and highlighting the applicability to other research and clinical practice without the opportunity for discussion.^53^,^56^,^57^ This transitioned to local email discussions allowing more interaction, but limited ability to readily organize and measure impact.^53^,^57^ There are now multiple pathways for running an online journal club.

One of the most commonly used resources for online journal clubs is Twitter. This platform has been used as the starting platform for numerous online journal clubs, as well as individuals, specialty organizations, journals, institutions, and events by providing instantaneous discussions with a diverse group of participants. 23,52,55,56,59,61–66 Participating in an online journal club also helps new Twitter users to rapidly build their own unique, high-quality, personal learning network.^56^

Twitter has rapidly become the dominant source of medical blog traffic while providing a platform for journals to tweet links to their latest papers. This initiates the community peer-review process and may predict future paper citations.^56^ For many online journal clubs, Twitter discussions are curated, summarized, and then linked to the initial PubMed citation for the article.^23^,^52^,^56^ This curated commentary may also be submitted and published as an article commentary or letter to the editor.^67^ Disadvantages of Twitter include character limits and lack of an underlying organizational structure, making delayed asynchronous engagement more challenging.^52^,^54^,^64^ Furthermore, many educators and learners do not use Twitter as consumers, or feel comfortable engaging in Twitter conversations as participants.^52^ Ultimately, more in-depth and even private discussions may be conducted on linked blogs or other online platforms.^23^,^53^,^55^,^58^

The rapid expansion and affordability of high-quality online video teleconferencing platforms (e.g., Skype, Google Hangouts) has created the ability to rapidly share and record journal club discussions across the globe.^10^,^53^ In 2013 the Academic Life in Emergency Medicine (ALiEM) team partnered with the *Annals of Emergency Medicine* on a year-long pilot of a series of online journal clubs discussing select articles from the journal.^52^,^58^ This unique project extended across multiple, social media platforms, beginning with a live Google Hangout session with topic experts, followed by the creation of podcasts and blog posts from these discussions.^52^,^58^ These online journal clubs were linked to and promoted by multiple medical blogs and websites, resulting in a significantly broader reach than attainable with a local journal club, as demonstrated by page views, Twitter activity, and comments.^52^,^58^

Other researchers have demonstrated that a hybrid model of live event and online discussions provides an adaptive and feasible educational delivery method for clinicians with limited educational time, with one study finding a positive improvement in the use of evidence-based recommendations by clinicians.^68^ At many training programs, faculty and learners may be working clinically at multiple institutions limiting the attendance for in-person journal club sessions. Yang and colleagues described the successful use of video conferencing software to increase attendance at monthly journal clubs.^59^

Other platforms have been used successfully including the Wikipedia software to create the Wiki journal club (WikiJC) in 2011.^54^ WikiJC begins each online journal club with a landmark article selected by the WikiJC editors. WikiJC entries can be written and edited by any registered person. Typically, the sections are authored by two people, with subsequent fact checking performed by the WikiJC editorial team. After this, the journal club discussion is disseminated through the website, email, and Twitter. WikiJC entries progress collaboratively from incomplete to a published status over an average of three-to-four weeks. With nearly 300 articles, WikiJC has become one of the most prolific online journal clubs to date.^23^,^54^

Suggested keys to success for a new online journal club include starting discussions on a single platform (e.g., Twitter) and then sharing the curated discussions on a blog or website; providing live discussions with the educator or author, followed by subsequent replies to learner comments across platforms; focusing on an initial small community of learners; ensuring that psychological safety is always ensured even after erroneous comments are posted; and tracking local participation and engagement.^23^,^52^,^53^,^58^

One study of general surgery residents found that an online journal club using a combination of email, Facebook, and Twitter was very well received by learners, noting that it was easy to participate in, helpful in keeping up with the latest literature, and valuable for developing critical appraisal skills.^67^ Learners found it significantly preferable to traditional journal club, and the authors noted that the online journal club led to the successful publication of eight article commentaries.^67^ Similar data were found by several other authors, noting improved learner satisfaction;^25^,^57^,^61^ however, more data are needed on higher level learning outcomes.

**Best Practice Recommendations:**

1. Consider using an online journal club format to increase participation and overcome the limitations of time, geography, and limited local membership (Level 4).
2. Before creating an online journal club, review successful, established online journal clubs and use previously established, effective strategies (Level 5).
3. Starting a new online journal club requires a significant time and resource commitment by a dedicated team. Ensure that this is feasible at your institution prior to launching an online journal club. Consider joining an existing online journal club rather than creating a new one if resources are limited (Level 5).

## LIMITATIONS

It is important to consider several limitations with respect to this article. While we used multiple methods to identify potential relevant articles, it is possible that some articles may not have been identified by the current review; however, we used an inclusive search strategy, reviewing all articles in PubMed that populated in response to the keywords “journal club.” We also discussed this with topic experts, reviewed the bibliographies for all relevant articles, and reached out via multiple social media networks for further resources. Nonetheless, it is possible that some articles not published in PubMed or referenced in the included articles may have been missed by this search strategy.

Additionally, we based article selection upon relevance to the specific themes selected. The topic of journal clubs is extensive and only specific components deemed to be most relevant to the clinician educator were selected for review in this publication. We also did not assess journal club effectiveness, as this has already been studied elsewhere.^17^ Instead, the focus was on practical, evidence-based recommendations for creating and maintaining a journal club. Finally, while preference was given to data directly evaluating journal clubs in EM residency programs, the data were more limited for this arena. Therefore, when data specific to EM residency programs were not available, we used data from other medical residencies and fields as a surrogate.

## CONCLUSION

This paper provides an evidence-based review of the literature on the design and structure of journal clubs in residency education. Strategies for preparation, topic selection, implementation, and integration of technology are discussed along with recommendations for best practices. After reading this paper, it is the authors’ intention that readers will have new strategies and techniques for implementing and running a journal club at their home institution.

**CORD Best Practice Committee 2017–18**

**Michael Gottlieb, MD – Co-Chair**
  Rush University Medical Center
**John Bailitz, MD – Co-Chair**
  Northwestern University, Feinberg School of Medicine
**Jeremy Branzetti, MD**
  New York University/Bellevue
**Richard Byyny, MD**
  Denver Health Medical Center
**Molly Estes, MD**
  Stanford University
**Puja Gopal, MD**
  University of Illinois in Chicago
**Albert Kim, MD**
  Washington University in Saint Louis
**Andrew King, MD**
  The Ohio State University
**Melissa Parsons, MD**
  University of Florida - Jacksonville

## Figures and Tables

**Figure:**
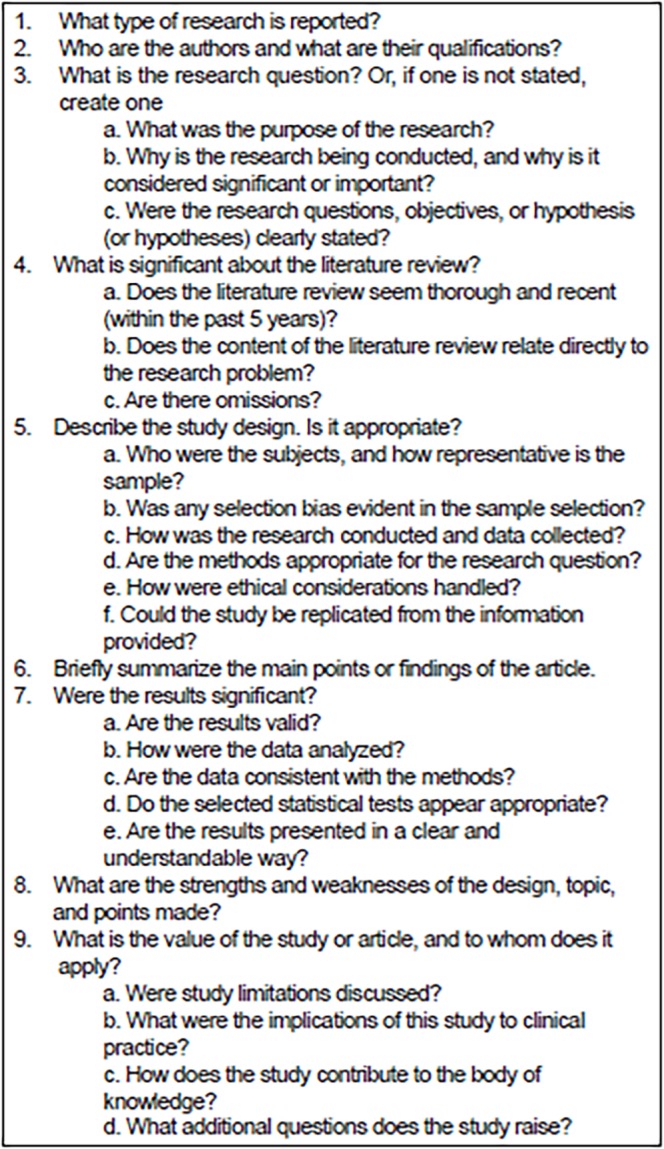
Example critical appraisal tool (adapted from Mazal and Truluck).^10^

**Table 1:** Oxford Centre for Evidence-based Medicine criteria.^8^

Level of evidence	Definition
1a	Systematic review of homogenous RCTs
1b	Individual RCT
2a	Systematic review of homogenous cohort studies
2b	Individual cohort study or a low-quality RCT*
3a	Systematic review of homogenous case-control studies
3b	Individual case-control study**
4	Case series or low-quality cohort or case-control study***
5	Expert opinion

*RCT,* randomized controlled trial.

* defined as <80% follow up;

** includes survey studies;

*** defined as studies without clearly defined study groups

**Table 2:** Objectives for journal club (adapted from Lee et al).^15^

Acquiring, disseminating, and applying new medical information Teaching and assessing critical appraisal skills for reading and writing a scientific paper Promoting lifelong learning skills in evidence-based medicine Improving reading habits Providing an interactive and social opportunity for peer-to-peer learning Improving small group participation, presentation, and communication skills Documenting practice-based learning and improvement in patient care

**Table 3:** Considerations for journal club topic selection.

New and upcoming literature Classic papers supporting current practice Articles generating clinical controversy Articles that are illustrative of specific methodologic techniques or biostatistical principles Manuscripts covered in blogs, podcasts, tweets, or other online sources Articles that align with other aspects of the curriculum being taught Articles reflecting original research rather than review articles or opinion pieces

**Table 4:** Four principles of adult learning theory.^34^

Adults need to be involved in the planning and evaluation of their instruction. Experience provides the basis for the learning activities. Adults are most interested in learning subjects that have immediate relevance and impact to their job or personal life. Adult learning is problem-centered rather than content-oriented.

## References

[1] Cushing H (1926). The Life of Sir William Osler.

[2] Paget S (1901). Memoirs and Letters of Sir James Paget.

[3] Linzer M (1987). The journal club and medical education: over one hundred years of unrecorded history. Postgrad Med J.

[4] Alguire PC (1998). A review of journal clubs in postgraduate medical education. J Gen Intern Med.

[5] Woods JR, Winkel CE (1982). Journal club format emphasizing techniques of critical reading. J Med Educ.

[6] Jouriles NJ, Cordell WH, Martin DR (1996). Emergency medicine journal clubs. Acad Emerg Med.

[7] Carpenter CR, Kane BG, Carter M (2010). Incorporating evidence-based medicine into resident education: a CORD survey of faculty and resident expectations. Acad Emerg Med.

[8] Phillips R, Ball C, Sackett D (2009). Oxford Centre for Evidence-based Medicine – Levels of Evidence.

[9] Hartzell JD, Veerappan GR, Posley K (2009). Resident run journal club: a model based on the adult learning theory. Med Teach.

[10] Mazal J, Truluck C (2014). Organizing and leading a journal club. Radiol Technol.

[11] Hohmann E, Tetsworth K (2016). Teaching residents: critical appraisal of the literature using a journal club format. Postgrad Med J.

[12] Linzer M (1987). The journal club and medical education: over one hundred years of unrecorded history. Postgrad Med J.

[13] Valentini RP, Daniels SR (1997). The journal club. Postgrad Med J.

[14] Moberg-Wolff EA, Kosasih JB (1995). Journal clubs. Prevalence, format, and efficacy in PM&R. Am J Phys Med Rehabil.

[15] Lee AG, Boldt HC, Golnik KC (2005). Using the Journal club to teach and assess competence in practice-based learning and improvement: a literature review and recommendation for implementation. Surv Ophthalmol.

[16] Greene WB (2000). The role of journal clubs in orthopedic surgery residency programs. Clin Orthop Relat Res.

[17] Harris J, Kearley K, Heneghan C (2011). Are journal clubs effective in supporting evidence-based decision making? A systematic review. BEME Guide No. 16. Med Teach.

[18] Al Achkar M (2016). Redesigning journal club in residency. Adv Med Educ Pract.

[19] Kattan JA, Apostolou A, Al-Samarrai T (2014). Beyond content: leadership development through a journal club. Am J Prev Med.

[20] Deenadayalan Y, Grimmer-Somers K, Prior M (2008). How to run an effective journal club: a systematic review. J Eval Clin Pract.

[21] Honey CP, Baker JA (2011). Exploring the impact of journal clubs: a systematic review. Nurse Educ Today.

[22] Stallings A, Borja-Hart N, Fass J (2011). New Practitioners Forum: Strategies for reinventing journal club. Am J Health Syst Pharm.

[23] Topf JM, Sparks MA, Phelan PJ (2017). The evolution of the journal club: rrom Osler to Twitter. Am J Kidney Dis.

[24] Green BN, Johnson CD (2007). Use of a modified journal club and letters to editors to teach critical appraisal skills. J Allied Health.

[25] Ahmadi N, McKenzie ME, MacLean A, Evidence-based reviews in surgery steering group (2012). teaching evidence based medicine to surgery residents - is journal club the best format? a systematic review of the literature. J Surg Educ.

[26] Burstein JL, Hollander JE, Barlas D (1996). Enhancing the value of journal club: use of a structured review instrument. Am J Emerg Med.

[27] Atzema C (2004). Presenting at journal club: a guide. Ann Emerg Med.

[28] Elnicki DM, Halperin AK, Shockcor WT (1999). Multidisciplinary evidence-based medicine journal clubs: curriculum design and participants’ reactions. Am J Med Sci.

[29] Sadeghi A, Biglari M, Nasseri-Moghaddam S (2016). Medical Journal club as a new method of education: modifications for improvement. Arch Iran Med.

[30] Inui TS (1981). Critical reading seminars for medical residents. Report of a teaching technique. Med Care.

[31] Joorabchi B (1984). A problem-based journal club. J Med Educ.

[32] Kitching AD, Ross JR (1992). Resuscitating the cardiology journal club. Can J Cardiol.

[33] Green ML, Ellis PJ (1997). Impact of an evidence-based medicine curriculum based on adult learning theory. J Gen Intern Med.

[34] Knowles M (1984). The Adult Learner: A Neglected Species.

[35] Kelly AM, Cronin P (2010). Setting up, maintaining and evaluating an evidence based radiology journal club: the University of Michigan experience. Acad Radiol.

[36] Edwards R, White M, Gray J (2001). Use of a journal club and letter-writing exercise to teach critical appraisal to medical undergraduates. Med Educ.

[37] Chakraborti C (2011). Teaching evidence-based medicine using team-based learning in journal clubs. Med Educ.

[38] Anzarut A, Martens B, Tredget E (2011). Improving journal clubs through the use of positive deviance: a mixed-methods study. Can J Plast Surg.

[39] Sidorov J (1995). How are internal medicine residency journal clubs organized, and what makes them successful?. Arch Intern Med.

[40] Shifflette V, Mitchell C, Mangram A (2012). Current approaches to journal club by general surgery programs within the Southwestern Surgical Congress. J Surg Educ.

[41] Pitner ND, Fox CA, Riess ML (2013). Implementing a successful journal club in an anesthesiology residency program. F1000Res.

[42] Mohr NM, Stoltze AJ, Harland KK (2015). An evidence-based medicine curriculum implemented in journal club improves resident performance on the Fresno test. J Emerg Med.

[43] Heiligman RM, Wollitzer AO (1987). A survey of journal clubs in U.S. family practice residencies. J Med Educ.

[44] Steele-Moses SK (2009). Developing a journal club at your institution. Clin J Oncol Nurs.

[45] Price DW, Felix KG (2008). Journal clubs and case conferences: from academic tradition to communities of practice. J Contin Educ Health Prof.

[46] Quinn EM, Cantillon P, Redmond HP (2014). Surgical journal club as a community of practice: A Case Study. J Surg Educ.

[47] Matthews DC (2011). Journal clubs most effective if tailored to learner needs. Evid Based Dent.

[48] Patelarou AE, Kyriakoulis KG, Stamou (2017). Approaches to teach evidence-based practice among health professionals: an overview of the existing evidence. Adv Med Educ Pract.

[49] McKeever S, Kinney S, Lima S (2016). Creating a journal club competition improves paediatric nurses’ participation and engagement. Nurse Educ Today.

[50] Ricciardi F, De Paolis LT (2014). A comprehensive review of serious games in health professions. Int J Comput Games Technol.

[51] Pitt MB, Borman-Shoap EC, Eppich WJ (2015). Twelve tips for maximizing the effectiveness of game-based learning. Med Teach.

[52] Lin M, Joshi N, Hayes BD (2017). Accelerating knowledge translation: reflections from the online ALiEM-Annals Global Emergency Medicine Journal Club experience. Ann Emerg Med.

[53] Chetlen AL, Dell CM, Solberg AO (2017). Another time, another space: the evolution of the virtual journal club. Acad Radiol.

[54] Plante TB, Iberri DJ, Coderre EL (2015). Building a modern journal club: the Wiki Journal Club experience. J Grad Med Educ.

[55] Chan TM, Thoma B, Radecki R (2015). Ten steps for setting up an online journal club. J Contin Educ Health Prof.

[56] Topf JM, Hiremath S (2015). Social media, medicine, and the modern journal club. Int Rev Psychiatry.

[57] Kawar E, Garcia-Sayan E, Baker-Genaw K (2012). Journal club 102: enhancing evidence-based medicine learning using a virtual journal club. J Grad Med Educ.

[58] Lin M, Sherbino J (2015). Creating a virtual journal club: a community of practice using multiple social media strategies. J Grad Med Educ.

[59] Yang PR, Meals RA (2014). How to establish an interactive eConference and eJournal club. J Hand Surg Am.

[60] Lizarondo L, Kumar S, Grimmer-Somers K (2010). Online journal clubs: an innovative approach to achieving evidence-based practice. J Allied Health.

[61] Udani AD, Moyse D, Peery CA (2016). Twitter-augmented journal club: educational engagement and experience so far. A A Case Rep.

[62] Dimov V, Randhawa S, Auron M (2009). The Utility of a Real-time Microblogging Service for Journal Club in Allergy and Immunology. American College of Allergy, Asthma & Immunology (ACAAI) 2009 Annual Meeting. Ann Allergy Asthma Immunol.

[63] Reich ES (2011). Researchers tweet technical talk. Nature.

[64] Thangasamy IA, Leveridge M, Davies BJ (2014). International Urology Journal Club via Twitter: 12-month experience. Eur Urol.

[65] Roberts MJ, Perera M, Lawrentschuk N (2015). Globalization of continuing professional development by journal clubs via microblogging: a systematic review. J Med Internet Res.

[66] Bayne CE, Cardona-Grau D, Hsieh MH (2017). Introducing the Pediatric Urology Journal Club on Twitter. J Pediatr Urol.

[67] Oliphant R, Blackhall V, Moug S (2015). Early experience of a virtual journal club. Clin Teach.

[68] Wilson M, Ice S, Nakashima CY (2015). Striving for evidence-based practice innovations through a hybrid model journal club: a pilot study. Nurse Educ Today.

